# Dual-Action Calcium Monoaluminate Enabled Room-Temperature Curing of Inorganic Phosphate-Based High-Temperature Adhesive

**DOI:** 10.3390/ma17184542

**Published:** 2024-09-15

**Authors:** Zhuo Dong, Lei Zhang, Ke Yang, Zhenggang Fang, Yaru Ni, Yang Li, Chunhua Lu

**Affiliations:** 1 State Key Laboratory of Materials-Oriented Chemical Engineering, College of Materials Science and Engineering, Nanjing Tech University, Nanjing 210009, China; 2 Jiangsu Collaborative Innovation Center for Advanced Inorganic Function Composites, Nanjing Tech University, Nanjing 210009, China; 3 Jiangsu National Synergetic Innovation Center for Advanced Materials (SICAM), Nanjing Tech University, Nanjing 210009, China; 4 College of Chemistry, Beijing University of Chemical Technology, Beijing 100013, China

**Keywords:** high-temperature adhesive, room-temperature curing, phosphate-based composite, calcium monoaluminate, nano-reinforcement

## Abstract

High-temperature adhesives find extensive application in diverse domains, encompassing repairs, production processes, and material joining. However, achieving their curing at ambient temperatures remains a formidable challenge due to the inherent requirement of elevated temperatures, typically exceeding 500 °C, for the curing reaction. To overcome this limitation, in this study, we developed a distinctive inorganic phosphate-based composite adhesive by incorporating dual-functional calcium monoaluminate (CA) into a traditional adhesive blend comprising Al(H_2_PO_4_)_3_ and MgO. This distinctive approach significantly diminishes the curing temperature, enabling it to occur at room temperature. Firstly, CA’s facile hydration reaction effectively scavenges surrounding water molecules, thereby accelerating the dehydration curing process of Al(H_2_PO_4_)_3_. Secondly, as hydration is an exothermic process, it locally generates heat around the Al(H_2_PO_4_)_3_, fostering optimal conditions for its curing reaction. Moreover, the adhesive’s strength is substantially bolstered through the strategic inclusion of Nano-Al_2_O_3_ (enhancing the availability of reaction sites) and Nano-SiO_2_ (improving overall stability). As a demonstration, the adhesive formulation with added CA containing 2% Nano-Al_2_O_3_ and 2% Nano-SiO_2_ achieved a remarkable tensile strength of 32.48 MPa at room temperature, underscoring its potential as an efficient solution for various practical adhesive applications. The adhesive prepared in this study harnesses the hydration properties of CA to absorb moisture and release substantial heat, introducing a novel method for ambient temperature curing. This development promises to broaden its applications in refractory materials, coatings, and equipment repair.

## 1. Introduction

**Figure 1:**
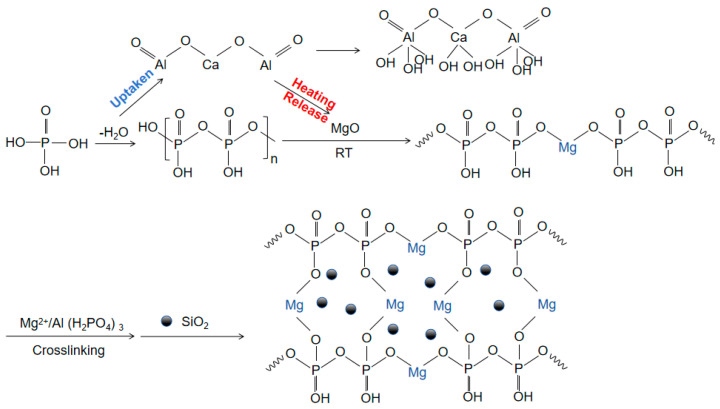
Mechanism diagram of hydration reaction of adhesive.

With the rapid development of industries such as aerospace and resource extraction, and the increasing use of special devices in extreme environments such as high temperature and high pressure [1,2,3,4,5,6], the demand for the performance of adhesive materials, especially ultra-high-temperature adhesive materials, continues to increase. Inorganic phosphate binders have broad application prospects and potential industrial value due to their simple structure, high bonding strength, and non-toxic and odorless properties [7,8,9]. However, the phosphate binder faces a challenge of curing at room temperature due to the typical high temperature of dehydration condensation reactions, which is approximately 500 °C, limiting the usability and practical application. Using curing agents to substitute other curing reactions that require lower reaction temperatures is now a common strategy [10,11,12]. MgO, through its ability to undergo a series of chemical reactions with the binder matrix, significantly lowers the curing temperature of the binder and is a commonly used curing agent. Taking Al(H_2_PO_4_)_3_ and MgO as an example, upon the addition of MgO, the binder system forms Mg(H_2_PO_4_)_2_·4H_2_O with superior bonding properties, significantly reducing the PO_4_^3−^ content in the solution and enhancing gel formation within the binder. This reaction is exothermic, with the generated heat facilitating the curing reaction. Subsequently, MgO encourages dehydration and condensation among phosphate molecules, leading to the formation of a comprehensive three-dimensional network structure. Mg^2+^ plays a pivotal role in this network structure, acting as a connecting bridge (Figure 1) [13,14,15,16]. J. A. Fernando [17] and Li Yizhen [18] lowered the curing temperature of the adhesive to 200 and 120 °C by controlling the P/Al ratio doped with MgO, but its curing temperature was still significantly higher than the ambient temperature.

As we know, calcium aluminate (CaO·Al_2_O_3_, CA) can easily react with water through a hydration process at room temperature, with the reaction equation being $CA+10H_{2}O={CAH}_{10}$ (T < 15 °C) [19,20]. Incorporating CA into the traditional adhesive blend of phosphate and MgO, on the one hand, can consume water molecules around the phosphate, promoting the dehydration process, but, on the other hand, the hydration of CA is an exothermic process, which generates heat around the Al(H_2_PO_4_)_3_, providing the necessary temperature conditions for the curing reaction without needing additional heating. Thus, this approach holds promise for achieving the curing reaction of phosphate high-temperature adhesive at room temperature [21,22,23,24,25].

Figure 1 shows a schematic diagram of the hydration reaction. Phosphoric acid is dehydrated by heating to form concentrated phosphoric acid. CA hydration absorbs water molecules generated by the dehydration condensation of Al(H_2_PO_4_)_3_, promoting the dehydration reaction. The horizontal and vertical binding between concentrated phosphoric acid and magnesium oxide forms a large molecular network structure through ‘-O-Mg-O-’. CA hydration releases a large amount of heat to promote the formation of the large molecular network structure. Nano-Al_2_O_3_ provides more nucleation sites [19,26,27,28]. Nano-SiO_2_, as an inert filler, does not participate in the reaction and plays a role in enhancing the thermal stability of the structure in the cross-linked network [29,30,31].

Herein, we developed a distinctive inorganic phosphate-based composite adhesive by incorporating the dual-functional CA into a traditional adhesive blend comprising Al(H_2_PO_4_)_3_ and MgO. The CA can simultaneously consume H_2_O and provide heat around the Al(H_2_PO_4_)_3_, thereby promoting the curing reaction. This distinctive approach significantly diminishes the curing temperature, enabling it to occur at room temperature. Moreover, the adhesive’s strength is substantially bolstered through the strategic inclusion of Nano-Al_2_O_3_ (enhancing the availability of reaction sites) and Nano-SiO_2_ (improving overall stability). As a demonstration, the adhesive formulation with CA containing 2% Nano-Al_2_O_3_ and 2% Nano-SiO_2_ achieved a remarkable tensile strength of 32.48 MPa at room temperature, underscoring its potential as an efficient solution for various practical adhesive applications.

## 2. Material and Methods

### 2.1. Materials and Manufacturing

**Figure 2:**
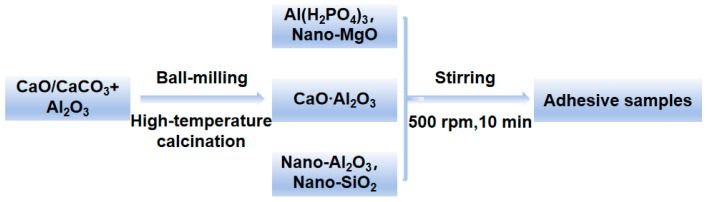
Process for preparing the inorganic phosphate adhesive.

Phosphate binders are mainly composed of matrix, curing agent, filler, etc. The matrix used in the adhesive is aluminum dihydrogen phosphate (Al (H_2_PO_4_)_3_) and calcium aluminate. During this process, magnesium oxide is used as the curing agent, and the fillers are composed of nano silica and nano alumina. Add CA to a beaker containing Al (H_2_PO_4_)_3_ at room temperature and stir mechanically at 500 rpm for 10 min to dissolve and mix evenly. Use a 400 μm coating rod to apply the adhesive onto the surface of the ceramic sheet (40 mm*40 mm*1 mm) for bonding, and cure at room temperature for 3 days. The process and formula are shown in Figure 2 and Table 1. The raw materials used in the experiment are shown in Table 2.

### 2.2. Tensile Strength Test

**Figure 3:**
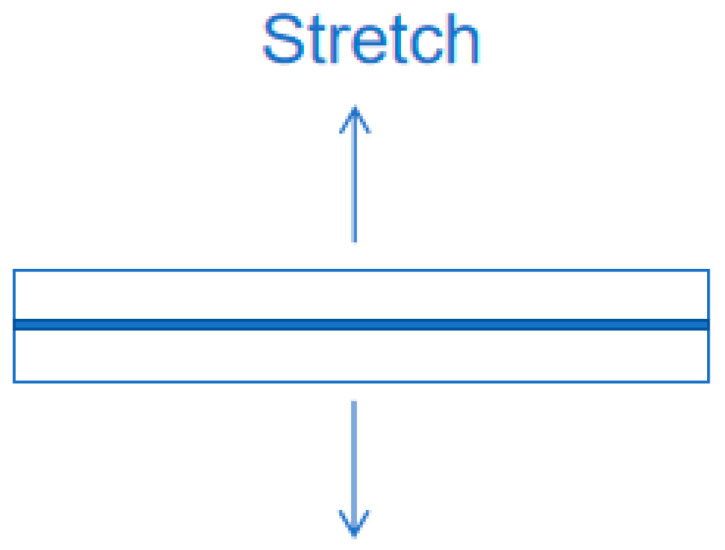
Schematic diagram of tensile strength test.

The bonding effect of phosphate binder is evaluated by testing the tensile strength of the binder bonded to ceramic tiles (30 mm*30 mm). After curing at room temperature and various environmental resistance tests, the binder is subjected to tensile testing using a universal testing machine to obtain the tensile strength (Figure 3). The test conditions are an inlet force of −1.25 N and a strain rate of 5 mm/min until the ceramic sample separates. Data analysis is based on the arithmetic mean of at least six measurements taken for each group of samples under the same conditions to ensure the accuracy of the experimental measurements. The calculation of tensile strength is shown in the following formula:

σ = F_m_/S_o_

σ—sample tensile strength (MPa); F_m_—maximum force (N) experienced during sample separation; S_0_—original cross-sectional area of the sample (mm^2^).

### 2.3. Thermogravimetric Differential Scanning Calorimetry (TG-DSC)

This study uses the TA-SDT650 synchronous thermal analyzer from TA Instrument from the United States (New Castle, DE, USA) to conduct thermal analysis on the cured powder of phosphate binder, determining the complete curing temperature of phosphate binder and characterizing its thermal behavior. The test temperature was raised from room temperature to 1200 °C, with a heating rate of 10 °C min^−1^, and the atmospheric conditions were an air atmosphere.

### 2.4. X-ray Diffraction Analysis (XRD)

This study analyzes the phase changes of synthesized calcium aluminate and the solidified binder using an ARLX’TRA X-ray diffractometer from American thermal power company (Thermo Fisher Scientific, Waltham, MA, USA). The testing conditions are Cu target K α ray wavelength λ = 0.15046 nm, scanning rate of 10° min^−1^, and 2θ angle ranging from 10° to 80°. We collected XRD data of the products using Crystallographica Search-Match 3.1 and Jade 5.0 analysis software to analyze and process the measured data.

### 2.5. X-ray Photon–Electron Spectroscopy (XPS)

We examine the alterations in binding energy following the incorporation of nanoparticles, thereby analyzing the progression of the hydration reaction. Place the sample to be tested into the Kratos AXIS Ultra DLD electron spectrometer from Thermo Fisher Scientific (Waltham, MA, USA) to begin X-ray photoelectron spectroscopy testing. Irradiate with X-rays, adjust the electron detector, calibrate the beam intensity and energy position of the X-rays, and collect data. Conduct qualitative analysis on the surface elements of the test sample based on the obtained data, including the valence state of metal elements, the bonding structure of metal or non-metal elements, etc. The binding energy in the measurement results was corrected using the C1s (284.80 eV) binding energy.

### 2.6. Scanning Electron Microscopy Analysis (SEM)

This study characterizes the microstructure of the cured calcium aluminate powder and phosphate-based composite coating using a JSM-IT200 scanning electron microscope from Nippon Electric Corporation (Minato City, Tokyo). Before testing, the powder sample is dispersed on the silicon wafer and dried and coated with gold spray. We then analyze its constituent elements through EDS spectrometer.

## 3. Results and Discussion

**Figure 4:**
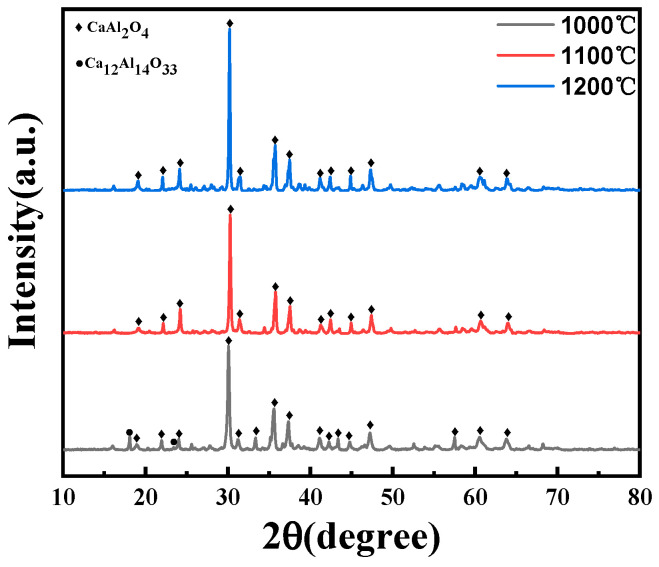
XRD patterns of samples synthesized at different sintering temperatures.

From Figure 4, when the calcination temperature is at 1100 and 1200 °C, the diffraction peak of the product is the pure CA phase, mainly because the Ca_12_Al_14_O_33_ phase begins to decompose at 900 and 1000 °C, and the following reaction occurs:

(1) $${Ca}_{12}{Al}_{14}O_{33}=Ca{Al}_{2}O_{4}+{Al}_{2}O_{3}$$

The reaction produces the CaAl_2_O_4_ and Al_2_O_3_ phases. At a heat treatment temperature of 1000 °C, the decomposition reaction is incomplete, and distinct Ca_12_Al_14_O_33_ diffraction peaks are still observable within the XRD diffraction patterns. Nevertheless, at temperatures of 1100 and 1200 °C, the decomposition reaction is complete, and the phase composition is dominated by CA.

**Figure 5:**
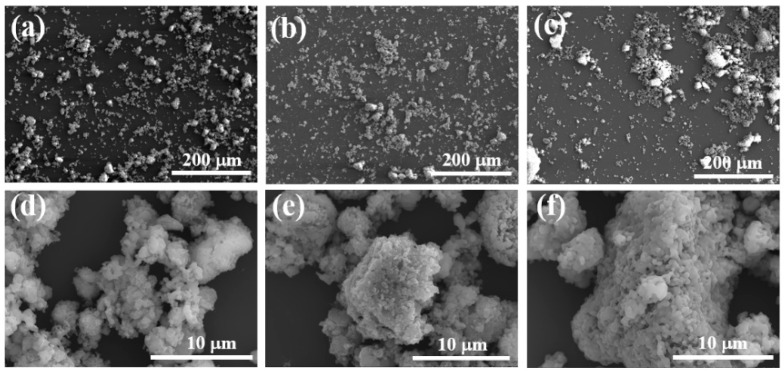
SEM images of synthetic samples at different sintering temperatures: (**a**,**d**) 1000 °C; (**b**,**e**) 1100 °C; (**c**,**f**) 1200 °C.

At a heat treatment temperature of 1000 °C, blurred grain boundaries are observable, with some grains undergoing phase transformation, suggesting they have not yet reached a state of complete growth (Figure 5d). At 1100 °C, the grains are well developed, with smaller size and more uniform distribution (Figure 5e). At 1200 °C, excessively high temperatures result in increased particle size and a non-uniform grain structure (Figure 5f). Consequently, 1100 °C is chosen as the heat treatment temperature for CA.

When only CA and Al(H_2_PO_4_)_3_ are used, the cured sample’s adhesive surface appears powdery upon peeling, suggesting an unstable internal structure. As the CA content rises, the powdering of the adhesive worsens. This could be because the high CA content swiftly depletes the phosphate adhesive’s moisture, triggering hydration reactions that disrupt the phosphate’s three-dimensional network, leading to reduced adhesive efficacy, weakened ceramic bonding, and resultant ceramic detachment. Therefore, CA needs to be used in conjunction with other curing agents.

### 3.1. Thermal Properties

**Figure 6:**
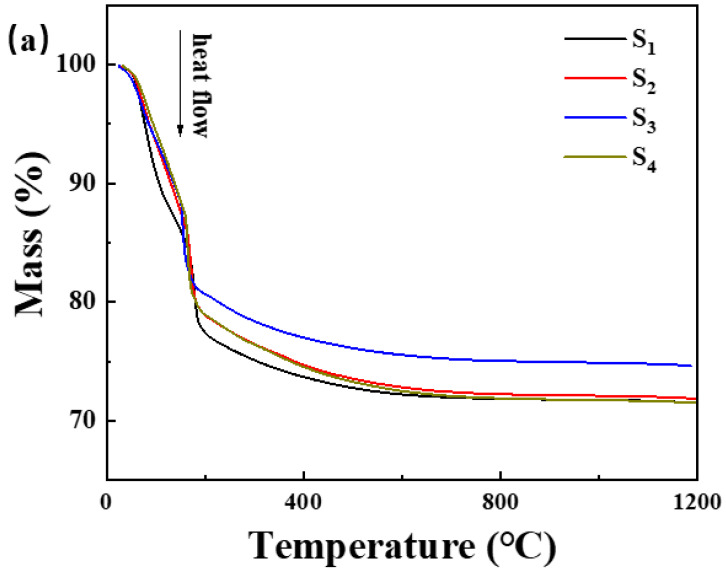

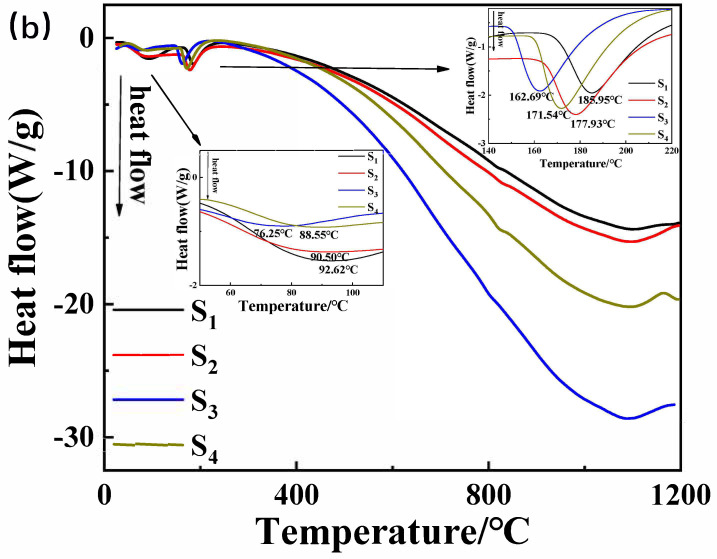
(**a**) Thermogravimetric curve and (**b**) differential scanning calorimetry curve of the samples in Table 1.

DSC curves of the adhesives with various curing promoters are shown in Figure 6.

**Figure 7:**
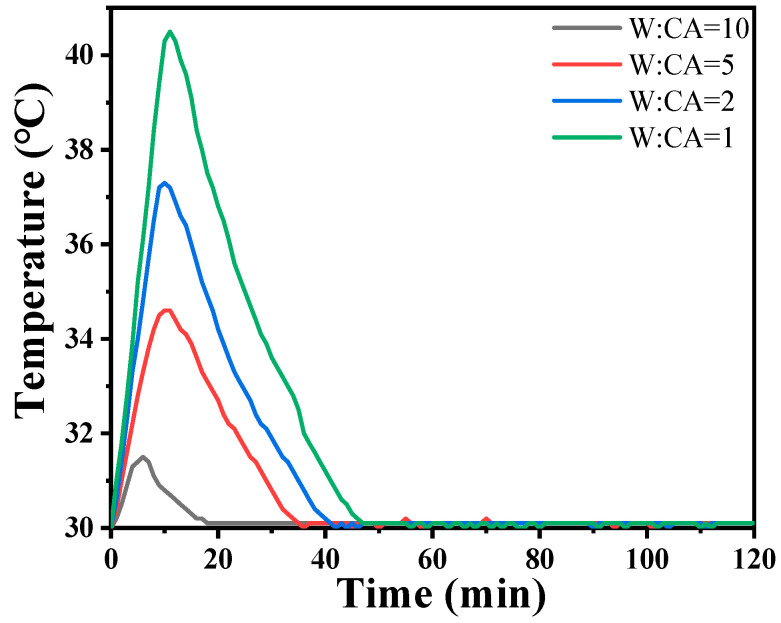
Hydration heat curves of CA solution at 30 °C.

The incorporation of Nano-MgO alone does not allow the phosphate reaction mixture to set at room temperature. However, the inclusion of CA enables the absorption of moisture within the mixture, elevates its internal temperature, and facilitates the solidification of phosphate. In Figure 7, we prove that the hydration of CA promptly liberates a significant amount of heat. When the mass ratio of water to CA is 10:1, the temperature peaks at an increase of 1.5 degrees Celsius within 8 min. However, when the mass ratio of water to CA was 1:1, the temperature soared to a maximum of 10.5 degrees Celsius within 9 min. All other samples can be cured. The addition of nanoparticles increases the reaction sites and connection points. To determine the curing reaction process of phosphate-based composite binders, four sets of samples were ground into powder and subjected to thermogravimetric differential thermal analysis. The test results are shown in Figure 7. It can be seen from Figure 6 that there are two obvious endothermic peaks on the DSC curves of the four groups of binder samples with different inorganic fillers added, corresponding to the preliminary curing peak and the complete curing peak of the phosphate-based composite binder, respectively. The preliminary and complete curing peaks of binders without inorganic fillers (S_1_) were 92.62 and 188.95 °C; the preliminary and complete curing peaks of binders with 4 wt.% Nano-Al_2_O_3_ (S_2_) were 90.50 and 177.93 °C; the preliminary and complete curing peaks of binders with 4 wt.% Nano-SiO_2_ (S_3_) were 76.25 and 162.69 °C; and the preliminary and complete curing peaks of binders with 2 wt.% Nano-Al_2_O_3_ and Nano-SiO_2_ (S_4_) were 88.55 and 177.93 °C. It is worth noting that after adding inorganic fillers, the curing temperature significantly decreases, and the most significant decrease in curing temperature occurs when nano alumina is added. Due to the acidic nature of phosphates and the higher alkalinity of nano alumina compared to silica, alumina can serve as both an inorganic filler and a curing agent to promote the curing of binders. Therefore, adding nano alumina will generate strong acid–base reactions, promote the curing process, and further reduce the curing temperature.

In summary, the TG-DSC curve indicated that the incorporation of CA absorbed moisture during the reaction, elevated the internal temperature, and facilitated the solidification of phosphate, thus lowering the curing temperature of the binder. Nano-Al_2_O_3_ and Nano-SiO_2_ underwent robust acid–base reactions, accelerating the curing process and further reducing the curing temperature.

### 3.2. XRD Patterns of the Specimens

**Figure 8:**
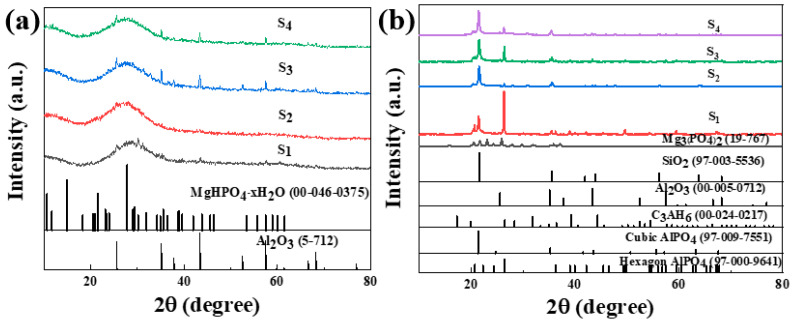
XRD patterns of phosphate-based composite binder before (**a**) and after heat treatment (200 °C for 2 h) (**b**).

Through the TG-DSC analysis mentioned above, it was determined that the complete solidification peak of the sample is around 200 °C. The sample was heat-treated at 200 °C for 2 h in a tube furnace and cooled down with the furnace. The XRD test was conducted on the original sample and heat-treated sample. The test results are shown in Figure 8.

**Figure 9:**
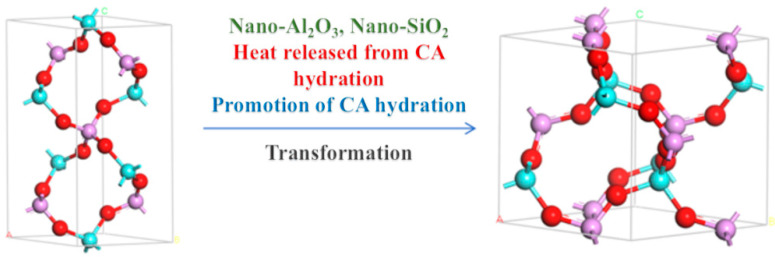
Crystal transformation during heat treatment process.

Figure 8a shows the XRD pattern of the phosphate-based composite adhesive after curing at room temperature for 5 days. After natural curing at room temperature for 5 days, a large hump appeared between 20 and 35°. The sample doped with a small amount of Nano-Al_2_O_3_ exhibited an incomplete reaction Al_2_O_3_ (ICDD-PDF 5-712) diffraction peak, and the incomplete reaction Al_2_O_3_ diffraction peak increased with an increase in the doping amount. The diffraction peak of MgHPO_4_∙xH_2_O (00-046-0375) appears in the sample due to the high activity of the reaction between MgO and the aluminum phosphate matrix. In addition, the system contains a small amount of CA hydration products, CAH_10_, C_2_AH_8_, and C_3_AH_6_ diffraction peaks, while other components exist in amorphous form. Figure 8b shows the XRD pattern of the phosphate-based composite adhesive after heat treatment. After heat treatment at 200 °C, the hump between 20 and 35° disappeared, and the samples were in a fully cured state. The hydration reaction of CA was complete in the four samples, and only the diffraction peak of the final product C_3_AH_6_ of CA hydration could be seen in XRD, indicating that the intermediate products CAH_10_ and C_2_AH_8_ were completely converted to C_3_AH_6_, with the strongest diffraction peak in S_1_. This is mainly because its CA content is the highest, resulting in the highest content of the hydration product C_3_AH_6_. The acceleration of the CA hydration process led to an increase in the reaction temperature in the system, accelerated the consumption of water in the system, and, thus, promoted the curing of the phosphate binder. Diffraction peaks of hexagonal AlPO4 and Mg_3_(PO_4_)_2_ of S_1_ were observed, indicating that Mg^2+^ entered the structure of the aluminum phosphate system and played a connecting role in the structure. With the addition of Nano-Al_2_O_3_, the diffraction peak of hexagonal AlPO_4_ decreases, while the diffraction peak of cubic AlPO_4_ relatively increases. Nano-Al_2_O_3_ promotes the transformation of hexagonal AlPO_4_ generated by the reaction into more stable cubic AlPO_4_ (Figure 9). Observing S_2_ and S_4_, in addition to the diffraction peaks of reaction-inert Nano-SiO_2_, Nano-SiO_2_ can also promote the transformation of hexagonal AlPO_4_ to cubic AlPO_4_ generated by the reaction. The addition of inorganic fillers can provide a large number of reaction sites, promoting the transition of the system to a more stable crystal phase. Therefore, both Nano-Al_2_O_3_ and Nano-SiO_2_ can serve as curing agents for phosphate binders to a certain extent, promoting the curing process of phosphate. Meanwhile, Nano-Al_2_O_3_ can accelerate the formation of CA hydration products, resulting in higher crystallinity of its hydration product C_3_AH_6_.

To ascertain the hydration process of the binder reaction, the binder reaction was initially accelerated through heat treatment. XRD results indicated that when only CA and Nano-MgO were doped, the product was completely transformed into C_3_AH_6_, with diffraction peaks of hexagonal AlPO_4_ and Mg_3_(PO_4_)_2_ of S_1_ observed. It was concluded that the accelerated CA hydration process elevated the system’s reaction temperature, thereby accelerating water consumption and enhancing the curing of phosphate binders. As seen in S_2_ and S_4_, the addition of nano fillers further facilitates the transformation of the reaction-formed hexagonal AlPO_4_ to cubic AlPO_4_. This is attributed to the numerous reaction sites provided by the inorganic fillers, which encourage the system’s transition to a more stable crystal phase. Consequently, the synergistic effect of CA and nano fillers enhances the binder’s stability.

### 3.3. Micromorphology

**Figure 10:**
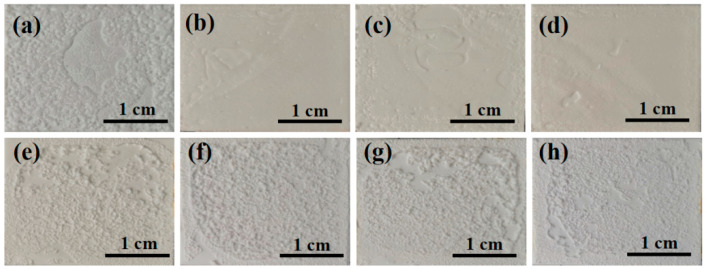
Surface morphology diagram of samples (**a**–**d**) after hydration and (**e**–**h**) after heat treatment.

Figure 10 shows the apparent morphology of the sample after hydration and heat treatment. It can be seen from the figure that at room temperature, with the addition of nano fillers, the binder structure becomes denser, resulting in higher tensile strength. Heat treatment can observe significant volume expansion of the adhesive, greatly reducing its tensile strength.

**Figure 11:**
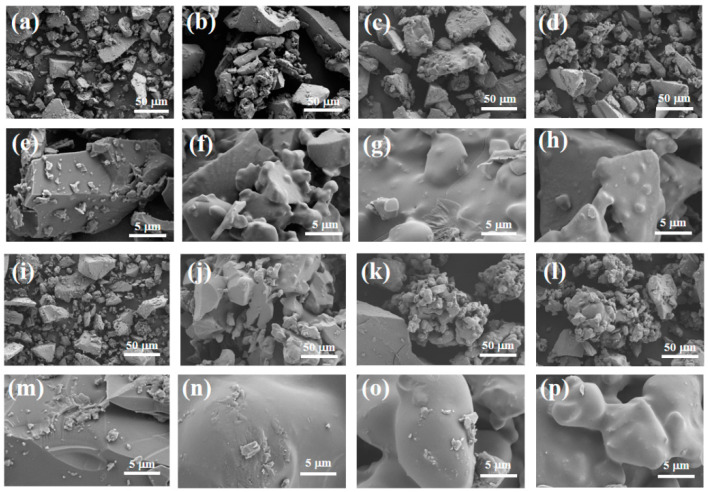
Scanning electron microscopy of samples (**a**–**h**) after hydration and (**i**–**p**) after heat treatment.

Figure 11 shows the surface microstructure of the adhesive at a magnification of 500 and 5000 times. It can be clearly observed from Figure 11e,m that there are many exposed particles on the surface of the adhesive, and there are cracks in S_1_. The surface quality of the adhesive is relatively rough. This is mainly because the CA content added by the binder is relatively high, resulting in more moisture in the coating, while the AP content is low, which is not enough to generate a large amount of the AlPO_4_ bonding phase to wrap the ceramic particles. When the coating is cured at high temperature, the liquid water evaporates due to heat and will be dispersed from between the exposed ceramic particles, leading to crack defects on the surface of the coating. However, the adhesive and surface in Figure 11f,h,n,p are flat and smooth, without obvious holes or cracks, and the density of the coating is significantly improved. This indicates that with an increase in the content of nano-SiO_2_, the particles can be better encapsulated, the adhesion between particles is improved, and the quality of the binder gradually improves. Therefore, it can be concluded that an increase in the content of nano-SiO_2_ helps to improve the surface quality of the binder.

**Figure 12:**
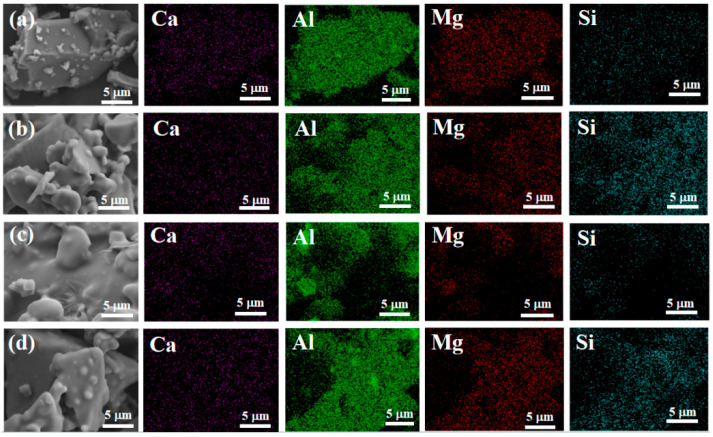
Energy-dispersive spectrometry of samples after hydration. ((**a**)-S_1_, (**b**)-S_2_, (**c**)-S3, (**d**)-S_4_).

Figure 12 shows the distribution of silicon elements on the surface of the sample. It can be seen that silicon elements are uniformly distributed in samples S_2_ and S_4_, indicating that the nano fillers are uniformly mixed into the binder.

Overall, at room temperature, as nano fillers were added to the binder, the structure became denser, and the tensile strength increased. After heat treatment, the adhesive structure underwent significant volume expansion, greatly reducing its tensile strength. Under scanning electron microscopy, we found that when only doped with CA, due to insufficient AP content, the adhesive reaction was too rapid, and there were still many unreacted exposed particles on the surface, which affected its tensile strength. However, after adding nano fillers, the surface was smoother. With an increase in nano fillers, the particles were better embedded, the adhesion between particles was improved, and the quality of the adhesive gradually improved. Therefore, it can be concluded that the increase in nano fillers helps to improve the surface quality of adhesives. Scanning the energy spectrum also revealed that the silicon element was uniformly distributed in samples S_2_ and S_4_, indicating that the nano fillers were uniformly mixed into the binder.

### 3.4. XPS

**Figure 13:**
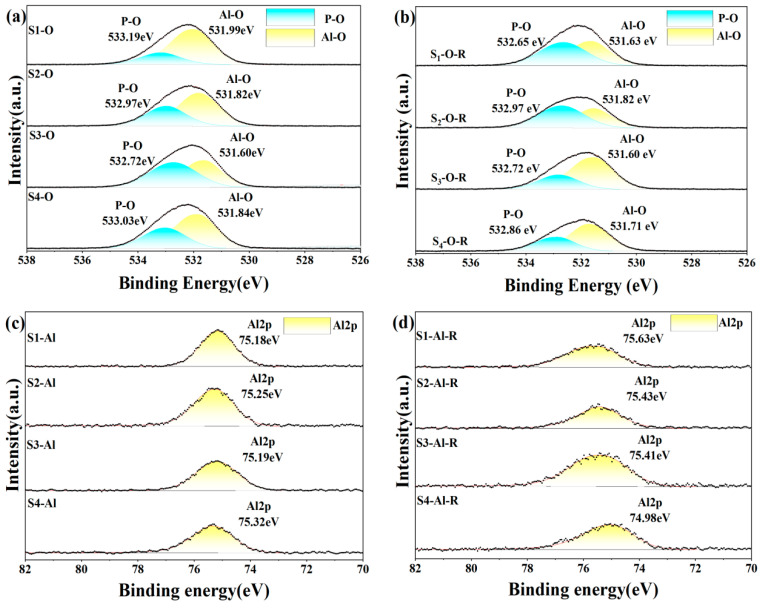

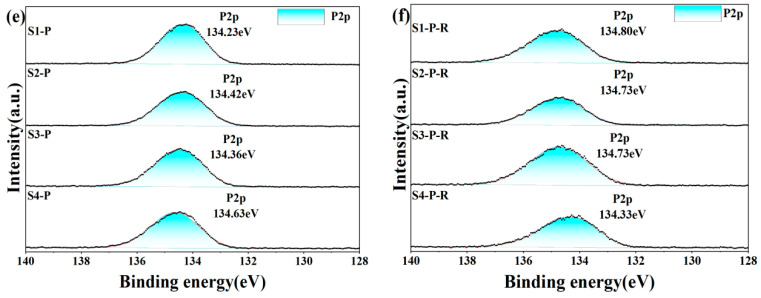
XPS spectrum of hydration samples before (**a**,**c**,**e**) and after heat treatment (200 °C for 2 h) (**b**,**d**,**f**).

To further understand how different inorganic oxides and phosphates bind to each other, we analyzed XPS narrow-spectrum scans of different elements before and after heat treatment of the sample. In the four groups of samples after five days of hydration, the Al2p and P2p spectra (Figure 13) showed that the binding energies of the doped inorganic nano fillers Al2p and P2p increased, the doped binding energy decreased, and the density of the outer electron cloud on the surface increased; the incorporation of nanoparticles promoted the hydration reaction. O1s spectra can be divided into groups of independent component peaks that overlap with each other from different groups. Using different nano-inorganic particles as a comparison sample, the O1s binding energy (531.82 eV) in the Al-O bond of the S_1_ sample was higher than that of the S_3_ sample (531.60 eV) in the O1s spectrum. In the O1s spectra of S_2_ and S_3_, the O1s binding energy (533.03 eV) in the P-O bond of S_4_ samples was higher than that of S_2_ and S_3_ samples, and the results showed that under the same conditions, the combined action of silica and alumina had a greater effect on the oxygen atoms on the P-O bond, and the combined action of silica and alumina promoted the curing process of phosphate. Among the four groups of samples heat-treated, it was found that the binding energy of S_1_ of the undoped nano filler increased the most after heat treatment, indicating that the heat treatment could accelerate the transformation of hexagonal AlPO_4_ to a more stable cubic AlPO_4_. It was found that the binding energies of Al2p and P2p in S_2_ and S_3_ samples increased but lower than that of the undoped S_1_ samples, indicating that the steric hindrance effect of nanoparticles (the steric hindrance effect primarily denotes the spatial obstruction arising from the proximity of certain atoms or groups within a molecule, and, with the incorporation of Nano-SiO_2_ and Nano-Al_2_O_3_, there is an elevation in activity at elevated temperatures, which sterically obstructs the reaction sites of the CA hydration, thus delaying the hydration reaction’s progression) hindered the progress of the hydration reaction under high-temperature conditions, and it was found that the binding energy of Al2p and P2p in S_4_ decreased, maybe because Nano-Al_2_O_3_ had high activity as a filler and reacted with Nano-SiO_2_ at high temperature, coupled with the steric hindrance effect, which seriously hindered the progress of the hydration reaction. The binding energy of the O1s spectrum was lower than that of the samples before heat treatment, which proved that the hexagonal crystal system AlPO_4_ changed to cubic AlPO_4_ after heat treatment.

### 3.5. Tensile Strength Test

**Figure 14:**
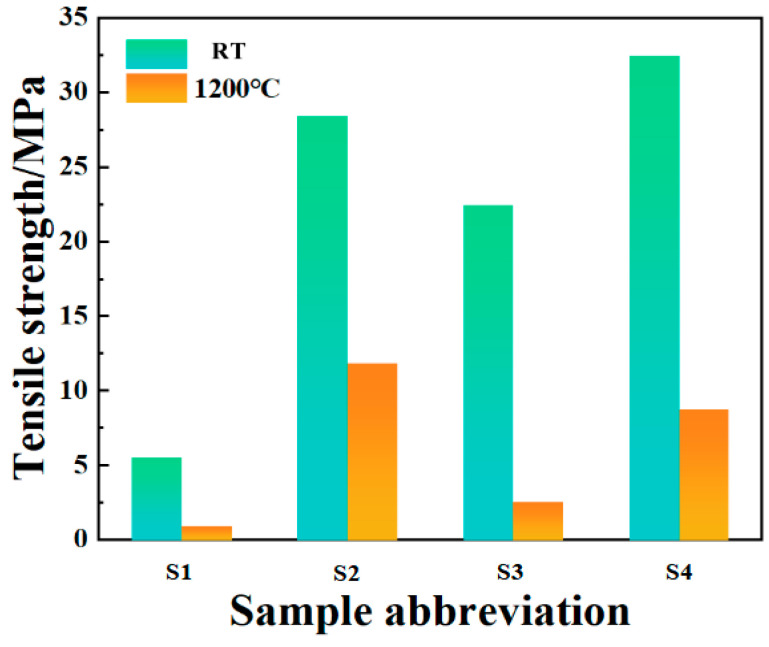
Tensile strength of bonded samples after room-temperature curing and high-temperature test.

Figure 14 shows the tensile strength of the bonded sample after room-temperature curing and high-temperature resistance testing. It can be seen that the bonding strength of the sample after room temperature curing is lower when no filler is added. This may be due to the high content of CA, excessive water consumption during hydration, and the hydration products filling the interior of the phosphate bonding agent to damage the network structure, resulting in a decrease in its strength. The addition of nano inorganic fillers as curing agents significantly improves the density and viscosity of the binder system. On the one hand, the addition of inorganic fillers can increase the density and viscosity of the binder system, provide a large number of reactive sites, and fill the pores of the binder to reduce the porosity, which helps the diffusion rate of reactants in the system. Rapid diffusion enables reactants to contact and react more quickly, thereby accelerating the curing process. On the other hand, Nano-Al_2_O_3_ can serve as a curing agent for phosphate binders and accelerate the formation of CA hydration products, further promoting the curing reaction. Among them, when the doping levels of Nano-Al_2_O_3_ and Nano-SiO_2_ are both 2 wt.%, the tensile strength is the highest, reaching 32.48 MPa. Compared to the values reported in the literature (7.56 MPa) [32], the tensile strength has significantly increased.

After curing at room temperature, the samples were subjected to high-temperature resistance tests at 1200 °C (Table 3). The tensile strength of the samples after removal is shown in the figure. It can be seen that the bonding ability of the samples without inorganic fillers is basically lost, indicating poor high-temperature resistance. However, after adding inorganic fillers, the tensile strength of the samples showed a certain degree of decrease. Among them, the samples in S_3_ and S_4_ experienced severe volume expansion, which may be due to the grain growth phenomenon of the particles under high-temperature conditions with the addition of Nano-Al_2_O_3_. Small grains migrate and rearrange at the interface to become larger grains. This grain growth causes the material to expand in volume, leading to the destruction of the network structure of the system by the fillers filled in the pores of the phosphate binder and further resulting in a significant loss of tensile strength in the system. Among them, when the inorganic filler is only Nano-SiO_2_ and the dosage is 4 wt.%, the decrease rate of the system’s tensile strength is the lowest, reaching 58.27%, and the tensile strength is 11.86 MPa at this time. Compared to the values reported in the literature (5.06 MPa) [32], the tensile strength has significantly increased.

Based on the TG-DSC and XRD analysis, it can be concluded that the curing mechanism of the aluminum phosphate-based composite adhesive is as follows: during the curing process of the aluminum phosphate composite adhesive, CA continuously consumes water in the hydration process of the system to generate hydration products of CAH_10_, C_2_AH_8_, and C_3_AH_6_, and the intermediate products continue to transform towards the stable state of C_3_AH_6_. The addition of inorganic filler Nano-Al_2_O_3_ can promote a higher degree of crystallization of C_3_AH_6_, which is conducive to the improvement in the adhesive strength of the system. CA continuously releases heat during the hydration process, which increases the temperature of the system and provides temperature conditions for the curing of aluminum phosphate. The addition of curing agent Nano-MgO can react with the adhesive. Mg^2+^ enters the interior of the binder and connects the chain like phosphate through ionic bonds, promoting the formation of a three-dimensional network structure and ultimately promoting the curing of the binder into a film. The addition of nano inorganic fillers fills the pores of the system, increasing the density and viscosity of the binder system while providing a large number of active sites for the reaction, promoting the curing reaction, reducing the curing temperature, and further improving the adhesive strength of the binder.

## 4. Conclusions

This study addresses the performance of an ultra-high-temperature inorganic phosphate adhesive that is cured at room temperature. It has been demonstrated that the hydration reaction of CA involves the absorption of substantial amounts of water and generates a significant amount of heat, thereby enhancing the curing process of the adhesive. Two types of nano fillers are incorporated to improve the adhesive’s performance, with Nano-Al_2_O_3_ increasing its reaction sites and Nano-SiO_2_ boosting its thermal stability. Ultimately, it was found that the adhesive formulation added CA containing 2% Nano-Al_2_O_3_ and 2% Nano-SiO_2_ achieved a remarkable tensile strength of 32.48 MPa at room temperature. After undergoing heat treatment at 1200 °C, the adhesive formulation fortified with 4% Nano-SiO_2_ in CA exhibited the least pronounced decline in tensile strength, with a reduction of merely 58.27%, resulting in a residual tensile strength of 11.86 MPa, underscoring its potential as an efficient solution for various practical adhesive applications. This strategy provides an efficient and reliable solution for a diverse range of practical high-temperature adhesive applications.

## Figures and Tables

**Table 1:** Curing promoter formulations (wt.%).

	Al(H_2_PO_4_)_3_	CaO·Al_2_O_3_	Nano-MgO	Nano-SiO_2_	Nano-Al_2_O_3_	State
S_0_	94	0	6			Uncured
S_1_	89	9	2			Cured
S_2_	89	5	2	4		Cured
S_3_	89	5	2		4	Cured
S_4_	89	5	2	2	2	Cured

**Table 2:** Experimental raw material.

Raw Material	Purity	Size	Company
CaO	AR	-	China National Pharmaceutical Group Chemical Reagent Co., Ltd. (Beijing, China)
Nano-Al_2_O_3_	99.5%	30 nm	Shanghai Aladdin Biochemical Technology Co., Ltd. (Shanghai, China)
Nano-MgO	99%	50–100 nm	Shanghai Aladdin Biochemical Technology Co., Ltd. (Shanghai, China)
Phosphoric acid	85%	-	China National Pharmaceutical Group Chemical Reagent Co., Ltd. (Beijing, China)
Al(OH)_3_	AR	-	Shanghai Meiruier Biochemical Technology Co., Ltd. (Shanghai, China)
Nano-SiO_2_	99.5%	15 nm	Shanghai Aladdin Biochemical Technology Co., Ltd. (Shanghai, China)

**Table 3:** Tensile strength of comparison sample and bonded samples after room-temperature curing and high-temperature test.

Sample	Room Temperature	1200 °C
S1	5.51 MPa	0.90 MPa
S2	28.41 MPa	11.86 MPa
S3	22.44 MPa	2.55 MPa
S4	32.48 MPa	8.71 MPa
Comparison sample [32]	7.56 MPa	5.06 MPa

## Data Availability

The original contributions presented in the study are included in the article, further inquiries can be directed to the corresponding authors.
